# Sequence-independent amplification coupled with DNA microarray analysis for detection and genotyping of noroviruses

**DOI:** 10.1186/s13568-015-0156-x

**Published:** 2015-11-10

**Authors:** Yuan Hu, Huijun Yan, Mark Mammel, Haifeng Chen

**Affiliations:** Northeast Region Laboratory, Office of Regulatory Affairs, Food and Drug Administration, Jamaica, NY USA; Department of Microbiology, Zhongshan School of Medicine, Sun Yat-sen University, Guangzhou, China; Division of Molecular Biology, Center for Food Safety and Applied Nutrition, Food and Drug Administration, 8301 Muirkirk Road, Laurel, MD 20708 USA

**Keywords:** Noroviruses, Sequence-independent amplification, DNA microarray, Detection, Genotyping

## Abstract

Noroviruses (NoVs) have high levels of genetic sequence diversities, which lead to difficulties in designing robust universal primers to efficiently amplify specific viral genomes for molecular analysis. We here described the practicality of sequence-independent amplification combined with DNA microarray analysis for simultaneous detection and genotyping of human NoVs in fecal specimens. We showed that single primer isothermal linear amplification (Ribo-SPIA) of genogroup I (GI) and genogroup II (GII) NoVs could be run through the same amplification protocol without the need to design and use any virus-specific primers. Related virus could be subtyped by the unique pattern of hybridization with the amplified product to the microarray. By testing 22 clinical fecal specimens obtained from acute gastroenteritis cases as blinded samples, 2 were GI positive and 18 were GII positive as well as 2 negative for NoVs. A NoV GII positive specimen was also identified as having co-occurrence of hepatitis A virus. The study showed that there was 100 % concordance for positive NoV detection at genogroup level between the results of Ribo-SPIA/microarray and the phylogenetic analysis of viral sequences of the capsid gene. In addition, 85 % genotype agreement was observed for the new assay compared to the results of phylogenetic analysis.

## Introduction

Noroviruses (NoVs) are recognized as the leading causative agents of outbreaks and sporadic cases of nonbacterial acute gastroenteritis across all ages in humans, resulting in more than 267,000,000 annual infections worldwide and over 200,000 deaths each year among children under 5 years old in developing countries (Noel et al. 1999; Patel et al. 2008; Donaldson et al. 2008). It is estimated that 21 million episodes of gastroenteritis are caused by NoVs annually in the United States (Scallan et al. 2001). NoVs are extremely infectious, and as low as 18 viral particles can cause disease (Teunis et al. 2008). The viruses most often transmitted through the fecal-oral route in semi-closed communities that favor person-to-person transmission, including schools, nursing homes, hospitals, restaurants and cruise ships. NoVs also spread by consumption of contaminated foods, making them leading causes of food borne disease (FAO/WHO 2007).

NoVs are the members of the genus *Noroviruses* in the family *Caliciviridae* (Pringle 1999). The viral genome is an approximate 7.5-kb positive single-stranded RNA that contains three open reading frames (ORFs) with a poly (A) tail at 3′ end (Jiang et al. 1990, 1993). The viruses are a broad range of enteric pathogens with great genetic and antigenic diversity (Wang et al. 1994; Green et al. 1995; Ando and Noel 2000). They segregate into 5 genogroups in which 3 genogroups (GI, GII, and GIV) are associated with human infection, with at least 8 genetic clusters in GI and 17 in GII (Zheng et al. 2006). Since human NoVs cannot be effectively cultivated in cell culture and laboratory animals, molecular methods have been increasingly used for their detection and characterization. Recently, reverse transcriptase polymerase chain reaction (RT-PCR) and subsequent genomic sequencing of the RT-PCR product have become the major means for detecting and characterizing the viruses. Generally, an efficient RT-PCR relies on finding the most conserved sequences across all virus genotypes to use as primers in order to efficiently amplify the maximum number of these diverse genetic variants. However, some sequence divergence has been observed even within the most conserved regions of the viral genome. Moreover, high level of genetic sequence variability of the viruses and continuous emergence of new virus variants (Siebenga et al. 2009) have complicated the design of robust universal primers for RT-PCR amplification of that many genetic variants for subsequent molecular analysis. Sequence-independent amplification methods (Wang et al. 2002; Berthet et al. 2008; Chen and Wang 2012) appear to be attractive alternatives to amplify diverse viruses for downstream applications including microarray analysis in that they do not require the prior sequence information of viral pathogens to guide to design virus-specific primers for amplification. This permits amplification of viral genomes from highly divergent viruses for which robust consensus primers focus on conserved regions are difficult to design.

We recently described RNA-based single primer isothermal linear amplification (Ribo-SPIA) of three diverse human enteric viruses including hepatitis A virus (HAV), NoV and coxsackievirus B2 (CXKV B2) from minute amount of starting viral RNAs without using any virus-specific primers. The amplified products were correctly identified by subsequent microarray analysis, displaying high level of reproducibility and fidelity in appropriate sensitivity ranges (Chen et al. 2013). In this study, we evaluated the utility of this sequence-independent RNA amplification method in combination with microarray analysis for detection and genotyping of the genetically diverse NoVs in fecal specimens.

## Materials and methods

### Viral RNA extraction

Twenty two fecal specimens from acute gastroenteritis were used in this study with approval of the FDA RIHSC. For control purpose, RNA of Norwalk virus (GI.1, accession #M87661) and a NoV #186 (GII.8, accession #HQ169542) were used as reference positive materials. Viral RNA was isolated using QIAamp viral RNA mini kit (Qiagen; Valencia, CA) per manufacturer’s instruction.

### Sequence-independent amplification of viral RNA

Viral RNA amplification was performed using a previously described Ribo-SPIA method (Richards et al. 2004) which is powered by NuGEN Ovation ^®^ pico WTA system (NuGEN Technologies; San Carlos, CA) following the manufacturer’s directions. The Ribo-SPIA includes 3 sequential reactions: first a reverse transcription reaction to generate first strand cDNA using a combination of random hexamers and poly-T chimeric primer; secondly a synthesis of DNA/RNA heteroduplex double strand cDNA with DNA polymerase; and thirdly a linear isothermal DNA amplification process in the presence of RNAse H, DNA polymerase, and a SPIA DNA/RNA chimeric primer. The final amplification product is single-strand cDNA (sscDNA) with sequence complementary to the original RNA.

### Quantification of amplified viral RNA by real-time RT-PCR

For the quantification of virus before and after Ribo-SPIA amplification, NoV GI- and GII- specific real-time RT-PCR (rRT-PCR) assays were performed on Norwalk and 186, respectively, in a SmartCycler instrument (Cepheid, Sunnyvale, CA). The GI- and GII-specific primers and probes were used in reactions as previously described (Kageyama et al. 2003). Amplification data were collected and analyzed with the SmartCycler system software.

### Microarray design, hybridization and data analysis

Virus detection and genotyping were assessed using the FDA_EVIR microarray chips described in previous study (Chen et al. 2011). The microarray, which was customer ordered to be manufactured by Affymetrix Inc (Affymetrix, Santa Clara, CA), interrogates approximately 91,000 25-mer oligonucleotide probes that derive from genomes of major human enteric viruses including NoV, HAV, CXKV, rotavirus, sapovirus, astrovirus, hepatitis E virus, and adenovirus. For each probe, there is a 23 base-pair overlap between consecutive probes within the same virus strain. The purified Ribo-SPIA products were treated with DNAse I (Invitrogen) at 37 °C for 1 min, and then were labeled with biotin-11-ddATP (PerkinElmer, Waltham, MA) in the presence of Terminal Transferase (Invitrogen) at 37 °C for 4 h. Microarray hybridization, washing, and staining were conducted following the standard procedure described in the GeneChip^®^ Expression Analysis Technical Manual (Affymetrix).

The microarray chips were scanned with GeneChip^®^ scanner (Affymetrix). The primary microarray data were analyzed with a script based on Affymetrix power tools as described previously (Chen et al. 2011). The results were considered positive for virus detection when the normalized hybridization signal intensities from the virus-specific array elements were three times greater than the background signal intensity. The background signal intensity was defined as the mean signal intensity for all the probe sets on the array.

### Direct sequencing and phylogenetic analysis

All virus samples used for the validation of the microarray genotyping results were sequenced directly. NoV capsid region was amplified by RT-PCR using primer sets of G1SK and G2SK described in a published literature (Kojima et al. 2002). HAV nested RT-PCR was performed on the sample 106 to amplify VP1/P2A junction region as described in previous studies (Robertson et al. 1992; Bower et al. 2000) using primer sets +2799 (5′ ATTCAGATTAGACTGCCTTGGTA 3′)/−3375 (5′ AGTAAAAACTCCAGCATCCATTTC 3′), and +2891 (5′ GGTTTCTATTCAGATTGCAAATTA 3′)/−3288 (5′ AACTTCATTATTTCATGCTCCT 3′) in the first and second around amplification, respectively. The RT-PCR products were purified and sequenced in both orientations using BigDye terminator chemistry on automated ABI Prism DNA analyzer (Applied Biosystems, Foster City, CA). Sequence analysis was conducted using ClustalX algorithm (Thompson et al. 1997), which was followed by phylogenetic analysis using neighbor-joining method as implemented in MEGA5 program.

### Nucleotide sequence accession numbers

The nucleotide sequences are deposited in NCBI GenBank under accession number KJ415779–KJ415798, and KJ437448.

## Results

### Quantification of amplified viral RNA

**Fig. 1:**
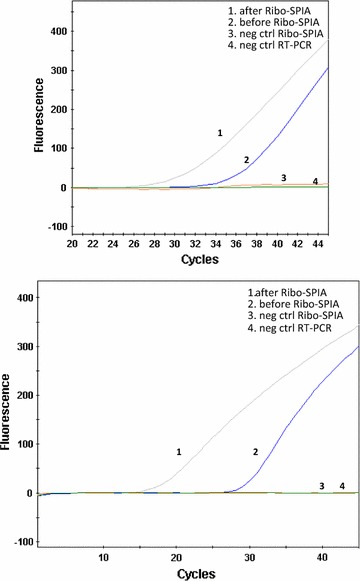
Ribo-SPIA method amplified both NoV GI and GII genomes. **a** NoV GI-specific real-time RT-PCR on Norwalk virus before (undiluted) and after Ribo-SPIA (10-fold dilution); ∆Ct = 9 which equals a 5120-fold increase in signal. **b** NoV GII-specific real-time RT PCR on NoV 186 before (undiluted) and after Ribo-SPIA (10-fold dilution). ∆Ct = 12 which equals 40960-fold increase in signal. Negative control for both Ribo-SPIA and PCR was nuclease free water

Quantitative analysis of amplified viral RNA was performed on Nowalk (GI) and 186 (GII), respectively, using rRT-PCR. Fold change was measured from ∆Ct value obtained from rRT-PCR results before and after Ribo-SPIA amplification, combined with dilution factor of 10 for each sample tested. As shown in Fig. 1, Ribo-SPIA amplification performed on Norwalk (Fig. 1a) and 186 (Fig. 1b) resulted in smaller Ct-values, indicating more target viral materials generated. Compared to non-amplification, approximately 5000-fold and 40,000-fold signal increases were achieved in Norwalk and 186, respectively. This process enabled amplifying both GI and GII NoV RNAs through the same amplification protocol without using multiple GI and GII-specific primer sets.

### Microarray analysis of clinical fecal specimens

**Fig. 2:**
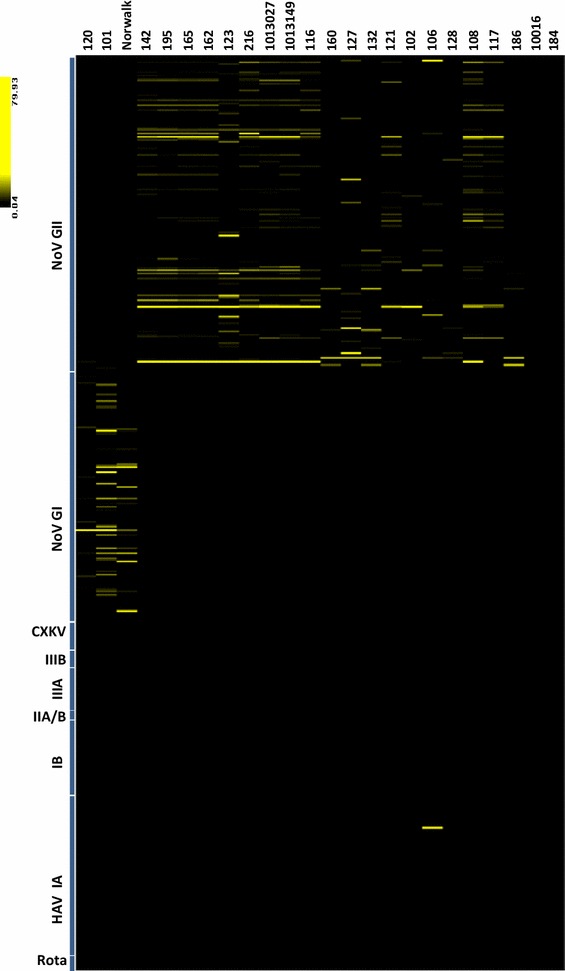
Microarray hybridization results from a blinded panel of 22 fecal samples and two positive reference materials including Norwalk (GI) and 186 (GII) strains. Hybridization signal intensity of each virus-specific probe element from the microarray is converted to color visualization scheme and depicted as a *vertical strip*. Signal intensity is reflected by the color of the stripe in which *black* indicates signal below threshold value of 3.0. Detection probe elements (*stripes*) are grouped by viral family of origin

Evaluation of discriminatory efficiency of the Ribo-SPIA/microarray system was accomplished by using a blinded panel of 22 RNA samples which were isolated from fecal specimens. Two positive reference strains of Norwalk and 186 were also included in the test. The results of microarray analysis are shown in Fig. 2. Of the 22 specimens tested, 20 gave patterns for specific hybridization to the probe elements derived from either NoV GI or GII genomic sequences, indicating positive results for clear NoV detection. The rest of two samples (10,016 and 184) serving as negative controls lacked detectable hybridization signal to all NoV-derived probes as well as the probes derived from other virus families, showing that no virus including NoV was detected in them. Two positive samples (120 and 101), together with a reference strain of Norwalk, hybridized strongly to the probes derived from NoV GI genome. Strong hybridizations to the NoV GII-derived probes were observed in the rest of positive 18 samples. By visual inspection, sample 142, 195, 165, 162, 216, 1013027, 1013149, 116 and 108 displayed similar hybridization pattern. So did sample 160 and 132 as well as reference strain 186. As shown in Table 1, a total of seven genotypes (GI.1, GII.2, GII.3, GII.4, GII.5, GII.8 and GII12) was identified, among which GII.4 was the most predominant genotype (11/20, 55 %). Furthermore, sample 106 tested positive for NoV GII was identified to have co-occurrence of HAV as subgenotype IA. The result here demonstrated that this amplification protocol coupled with microarray analysis was able to detect not only individual NoV but also co-occurrence of NoV and HAV present within the same sample.

**Table 1:** Comparison of the genotypic results between Ribo-SPIA/microarray and phylogenetic analysis

Ribo-SPIA/microarray									Phylogenetic analysis							
NoV								HAV^a^	NoV							HAV^a^
Genogroup^b^	GI	GII							GI		GII					
Genotype^c^	1	2	3	4	5	8	12	IA	1	8	1	2	4	5	8	IB
No. of specimens	2	1	1	11	2	2	1	1	1	1	2	1	11	2	2	1

^a^One fecal specimen was identified as having co-occurrence of NoV and HAV

^b^There was 100 % (20/20) concordance for positive NoV detection at genogroup level

^c^There was 85 % genotype agreement

### Phylogenetic analysis

**Fig. 3:**
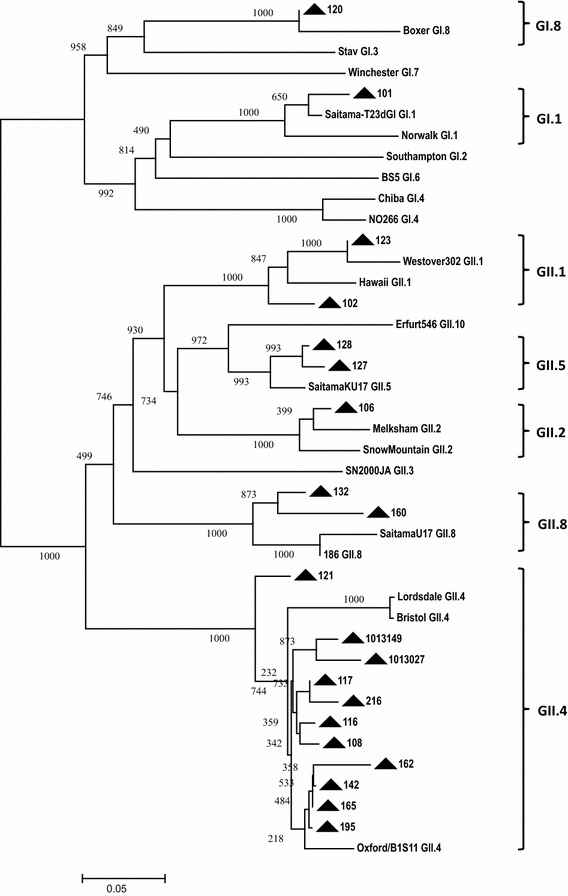
Phylogenetic dendrogram of NoV strains based on partial nucleic acid sequences of capsid region was generated using neighbour-joining method with ClustalX algorithm and MEGA5 program. *Numbers* on each branch indicate supporting bootstrap value of 1000 resampled data sets. NoV strains originating from this study are indicated with *black triangles*. Putative genotypes are indicated for each cluster

**Fig. 4:**
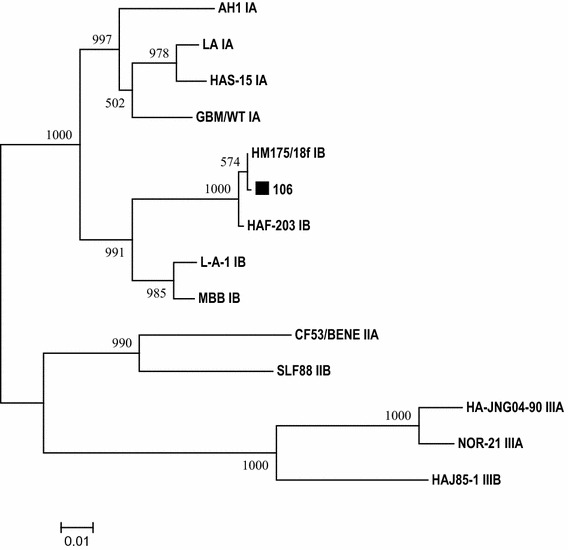
Phylogenetic dendrogram was constructed based on nucleic acid sequences of HAV VP1/P2A region of 15 strains using neighbour-joining method with ClustalX algorithm and MEGA5 program. *Numbers* on each branch indicate supporting bootstrap value of 1000 resampled data sets. HAV strain (106) obtained in present study was indicated with *black square*

The RT-PCR detected GI or GII NoV in 20 specimens except in 10,016 and 184. This was in line with the positive microarray results. With 20 NoV-positive samples, partial viral capsid genes were amplified by RT-PCR and sequenced. Based on the phylogenetic result, samples 120 and 101 were clustered to genotype GI.8 and GI.1, respectively. The remaining 18 samples were categorized into 5 GII genotypes including GII.4 (11), GII.8 (2), GII.1 (2), GII.5 (2) and GII.2 (1) as shown in Fig. 3. As a result, there was 100 % concordance for positive NoV detection at genogroup level between the results of microarray and phylogenetic analysis (Table 1). Three samples 120,123 and 102 which were genotyped as GI.1, GII.12 and GII.3, respectively, in microarray analysis were identified as GI.8, GII.1 and GII.1 in the phylogenetic result. Thus, 85 % (17/20) genotype agreement was observed between the results of Ribo-SPIA/microarray and phylogenetic analysis (Table 1). No statistically significant difference was detected between the two results (McNemar’s test; *P* = 0.2482). In addition, HAV RNA was also detected by nested RT-PCR in 106 which was NoV GII positive. Sequence comparison between the nested RT-PCR product with other HAV strains revealed that the virus displayed the highest sequence similarity to a subgenotype IB strain HM175/18f (Fig. 4).

## Discussion

Reverse transcription followed by PCR reaction with primer sets designed to amplify specific viral RNA regions is the method of choice to amplify human NoVs prior to downstream molecular analysis. However, high sequence variability of the viral agents posts a challenge to the design of robust universal virus-specific primer sets to amplify various virus variants. Recent studies described the use of multiple GI and GII-specific degenerate primer sets for rRT-PCR to detect a wide range of GI and GII NoVs (Kageyama et al. 2003; Kojima et al. 2002; Richards et al. 2004). Those degenerate primer sets targeted either ORF1-ORF2 junction or RNA-dependent RNA polymerase region encoded by ORF1of the viral genome, indicating their design still relied on the virus sequence knowledge. In present study, we sought to establish a universal sequence-independent amplification procedure suitable for the highly divergent infectious agent to prepare sufficient amounts of target nucleic acids for microarray analysis. By using Ribo-SPIA, both NoV GI and GII viral RNA could be run through the same amplification protocol without the need to design and use any virus-specific primers. Ribo-SPIA amplification resulted in ~5000-fold and ~40,000-fold increase in RT-PCR signal for reference strains of Norwalk (GI) and 186 (GII), respectively (Fig. 1). Since GI and GII have been found to contain at least 8 and 17 genotypes, respectively (Zheng et al. 2006), ideal diagnostic tests for NoV should display strong discriminatory power in detecting such a wide variety of NoV genotypes. In this study, a panel of 22 fecal specimens was used to assess the reactivity of the system. Of them, 20 samples were observed positive NoV detection on the microarray showing positive hybridization signals (Fig. 2). The clinical sensitivity of the Ribo-SPIA/microarray system, determined by comparison with the detection rate by virus-specific RT-PCR with the same specimens, was 100 % at genogroup level. The specificity of the system was calculated to be 100 % as well. This indicates that Ribo-SPIA is readily applicable to the amplification of multiple sample types of the viruses for microarray analysis.

In comparison to RT-PCR based assays, which require the design and optimization of virus-specific primers for target amplification and only provide presumptive results for the presence or absence of the queried viruses, this sequence-independent amplification method offers several advantages. It obviates the need for an assumption to guild testing suspected viral pathogens with multiple viral-specific primers to amplify targets present in a sample, thus does not require prior sequence knowledge of the viral agent for primer design. Furthermore, a number of viruses cause similar clinical symptoms which make it difficult to choose correct diagnostic analysis under certain clinical hypothesis. In such case, the lack of virus-specific primer sets enables detection of viral agents that might not be found with virus-specific RT-PCR assays. Sample 106 that was presumed negative for HAV infection went merely under NoV RT-PCR test initially. But the microarray analysis here revealed that a subgenotype IA HAV and a GII NoV were simultaneously identified in this sample after Ribo-SPIA amplification (Fig. 2). The presence of HAV was further confirmed as subgenotype IB by phylogenic analysis of the sequence of the HAV-specific RT-PCR product. This parallel detection of mixed agents present in a specimen would benefit a better understanding of etiology of viral disease. Although there was an identification discrepancy at subgenotype level between the microarray and phylogenetic results, the HAV was indeed identified as the same genotype I. The discrepant results could be attributed to cross-reactivity between the probes targeting IA and IB genomes due to high level of genetic sequence similarity shared by the two groups of subgenotype strains (Robertson et al. 1992). Certain levels of cross-reactivity between IA and IB of HAV have been observed in previous studies (Chen et al. 2011, 2013). Similar false genotypic identification of NoV was also observed in 3 specimens when comparing to the results obtained in phylogenetic analysis, although they all fell within the same genogroups. These could be associated with cross-hybridization due to a combination factors such as the level of sequence variability, viral load, and amplification efficiency, which have an impact to the cross-hybridization pattern of the viruses (Boriskin et al. 2004). Future refinement to the current probe design involving the selection of only those probes that convey the highest discriminatory value in identifying viruses may help address the cross-reactivity issue as described here.

This study represents the first effort to describe the application of Ribo-SPIA method to amplify the genetically diverse NoVs without using any virus-specific primers for microarray-based detection and genotyping. This sequence-independent amplification linked to DNA microarray analysis allowed identification of multiple NoV genotypes tested in current study. It is expected that this system will also be able to detect other NoV genotypes not examined here since the probe elements derived from those respective viral genomes have already been printed on the microarray chips. Moreover, the use of the sequence-independent amplification protocol eliminates the need for a priori genomic sequence knowledge of the viral agents and thus can extend the range of applications to other RNA viruses.
